# Socioeconomic inequalities in health among Swedish adolescents - adding the subjective perspective

**DOI:** 10.1186/s12889-017-4863-x

**Published:** 2017-10-23

**Authors:** Mikael Ahlborg, Petra Svedberg, Maria Nyholm, Antony Morgan, Jens M. Nygren

**Affiliations:** 1 School of Health and Welfare, Halmstad University, SE 301 18 Halmstad, Sweden; 2 Glasgow Caledonian University, Glasgow, UK

**Keywords:** Socioeconomic inequality, Self-rated health, Socioeconomic status, Adolescence, HBSC

## Abstract

**Background:**

Socioeconomic inequalities in adolescent health predict future inequalities in adult health. Subjective measures of socioeconomic status (SES) may contribute with an increased understanding of these inequalities. The aim of this study was to investigate socioeconomic health inequalities using both a subjective and an objective measure of SES among Swedish adolescents.

**Method:**

Cross-sectional HBSC-data from 2002 to 2014 was used with a total sample of 23,088 adolescents aged 11–15 years. Three measures of self-rated health (dependent variables) were assessed: multiple health complaints, life satisfaction and health perception. SES was measured objectively by the Family Affluence Scale (FAS) and subjectively by “perceived family wealth” (independent variables). The trend for health inequalities was investigated descriptively with independent t-tests and the relationship between independent and dependent variables was investigated with multiple logistic regression analysis. Gender, age and survey year was considered as possible confounders.

**Results:**

Subjective SES was more strongly related to health outcomes than the objective measure (FAS). Also, the relation between FAS and health was weakened and even reversed (for multiple health complaints) when subjective SES was tested simultaneously in regression models (FAS OR: 1.03, CI: 1.00;1.06 and subjective SES OR: 0.66, CI: 0.63;0.68).

**Conclusions:**

The level of socioeconomic inequalities in adolescent health varied depending on which measure that was used to define SES. When focusing on adolescents, the subjective appraisals of SES is important to consider because they seem to provide a stronger tool for identifying inequalities in health for this group. This finding is important for policy makers to consider given the persistence of health inequalities in Sweden and other high-income countries.

## Background

Existing socioeconomic inequalities in adolescent health can present future health challenges since they are known to predict inequalities in adult health and life expectancy [1]. Evidence for socioeconomic inequalities in adolescent self-rated health and well-being have been demonstrated in international studies [2, 3]. Cross-national comparisons have shown that these inequalities are greater in countries of lower national income and in those with higher income inequality [4]. While individual-level socioeconomic status (SES) has proven to be a strong predictor of adolescent self-rated health, macro-level determinants such as national income or income inequality, can partly explain cross-national differences [4–6]. Other research confirms micro-level factors, such as family, school or neighbourhood characteristics, to be associated with self-rated health among adolescents [7]. Still, it seems as if the country in which a child lives, contributes to the level of reported self-rated health. This could be explained by the cultural context within a country, which affects the influence of micro-level factors of SES on health, and thereby the level of self-rated health that adolescents report [7].

When focusing on socioeconomic inequalities in adolescent health within the Swedish context, there are some issues that should be addressed to increase our understanding of where Sweden stands today. As is well known, the social gradient in health occurs not only when comparing health outcomes between poor and wealthy, but along every step of social class [8]. Even though the gradient is stronger in countries of high income-inequality, it has also been found significant in the Swedish context comparing parental income and child health [9]. At an international level, Sweden has for a long time been one of the most equal countries regarding income. However, over the last few decades, OECD data shows that income inequality has grown at a faster rate in Sweden compared to other countries. For example, in 2012, the top 10% of income earners had 6.3 times higher average income than those in the bottom 10%. This compares to a 4 to 1 ratio during the early 1990s [10]. Research shows that increasing wealth only amongst the richest lowers the national gross domestic product (GDP) and that without active policy-making to favour groups of lower SES, accessibility to high quality healthcare and education is lowered [11]. Compared to Norway and Finland, countries that are comparable in levels of macro- and micro-SES [5, 12, 13], Sweden stands out by displaying a higher prevalence of self-rated health complaints (e.g. headache, abdominal pain or feeling low) among adolescents. Moreover, recent trend data (between 2010 and 2014) shows a significant increase in such complaints among adolescents in Sweden which is not seen in neighbouring countries [2, 13]. The percentage of adolescents that report high life satisfaction has also decreased in Sweden over the same time period and is lower than in Norway and Finland, especially among 15-year olds. Previous research has examined health inequalities in Swedish children, and found apparent differences by level of SES [14]. However, depending on what measures of SES and measures of health that are being investigated, there is great variation in strength of this association. For example, adolescents have difficulties responding adequately to some measures, such as parental occupation, which can pose a challenge for research when adolescents are the only source of information [15].

The Health Behaviour of School-aged Children survey (HBSC) has monitored the health of adolescents for over three decades and has today over 40 participating countries. Sweden has participated in the survey since 1985. A brief review of research based on data from the HBSC shows that there is methodological variation in the approach taken to measure self-rated health. Most commonly, self-rated health complaints (e.g. headache, abdominal pain, feeling low) are used as an outcome measure either to capture prevalence of health complaints in clusters, exemplified by Ottová-Jordan et al. [16], or as Elgar et al. have illustrated, in investigative research on health determinants [17]. Health complaints are primarily assessed by the HBSC-symptom checklist (SCL), which includes eight psychosomatic symptoms. Researchers have however stressed the importance of not merely considering health from a deficit approach, but to also include feelings and attitudes towards health and life in general [18, 19], which are absent in the symptom checklist. For this reason, overall life satisfaction and general health perception, items in the HBSC questionnaire, are often used in combination with health complaints in order to capture both self-rated health and subjective well-being. These measures of health together, more thoroughly described in previous research [20], cover both the traditional, medical approach to adolescent health as well as aspects of well-being.

### Measures of socioeconomic status

Previous research has shown that the use of more than one measure of SES is both relevant and necessary when investigating socioeconomic health inequalities among adolescents [21, 22]. This is not dissimilar to research relating to adult SES, which has traditionally been assessed using a range of measures including: level of education; occupational status; and income. However, in the case of adolescent research, reliable information relating to SES is harder to collect. The Family Affluence Scale (FAS) was developed by the HBSC-network to enable researchers to achieve reliable information on family SES, albeit self-reported through adolescents [23]. Information obtained by FAS, has proven to be a reliable proxy for objective family SES [24]. That said, in more recent years SES has also been assessed using a more subjective approach [25]. Subjective SES is usually defined as a person’s perception of his/her social standing in reference to other members of a group [26]. The HBSC survey offers an example of this through its “perceived family wealth” measure. It is used frequently in analytic studies and has previously shown reliability in predicting inequalities in health [3, 25, 27]. Given, the imperative in Sweden to find ways of reducing socioeconomic inequalities amongst adolescents, there is increasing need for better measures so that inequalities can be better understood [14]. It is therefore necessary and interesting to investigate the relevance and benefit of adding a subjective measure of SES to analyses so that a more rounded understanding of inequalities can be observed.

Country specific understandings of socioeconomic inequalities in adolescent health are required to support appropriate and context focused policy action. This study aims to investigate socioeconomic inequalities in adolescent health in a Swedish context over time. It uses two measures of SES, as well as multiple assessments of health. This will allow a more detailed assessment of the relationships, as well as highlighting the complexities of methodology in adolescent health research.

## Methods

### Sample and procedure

This study investigated socioeconomic inequalities in adolescent health by using cross-sectional data from the Swedish HBSC-survey. The HBSC study is carried out every 4 years in collaboration with the World Health Organization. The purpose of the HBSC is to monitor the health and health behaviours of adolescents aged 11 to 15 years, by gathering cross-sectional data through a self-reported questionnaire. The original data used as part of this study was collected following the HBSC standardized international research protocol where participating school classes are randomly selected through a two-stage cluster sample [13, 28]. The sample was evenly distributed across gender and age (11, 13 and 15 years). This study is based on data from 2002 (*n* = 3926), 2006 (*n* = 4415), 2010 (*n* = 6880) and 2014 (*n* = 7867), giving a total sample of *n* = 23,088.

### Measures

Three measures of self-rated health were chosen as dependent variables: subjective health complaints, life-satisfaction and health perception. Subjective health complaints were assessed using the HBSC-symptom checklist (SCL) [29]. The measure reflects two dimensions from which health-complaints originate, somatic and psychological [30]. Students were asked how often they have experienced the following complaints over the past 6 months: headache, abdominal pain, backache, dizziness (somatic), feeling low, irritability/bad temper, feeling nervous, and sleeping difficulties (psychological). Response alternatives ranged from “seldom or never” through to “about every day”. Following recommendations from previous research, the SCL was summarised into a composite 8-item measure [31]. Response categories were grouped into “at least two complaints, more than once a week” vs. “less” to produce a dichotomous dependent variable. Adolescents in the former group were identified as having multiple health complaints (MHC). This cut-off point is used commonly to show a level of recurrent MHC that is likely to impair everyday functioning [2, 19, 32, 33]. Cantril’s ladder was used to measure life satisfaction. Respondents were shown a ladder with steps ranging from 0 to 10 where 0 equals the worst possible life and 10 the best possible life. It is easily understood and has shown high reliability among adolescents in previous research [34]. The mean value (7.59) was used to establish a threshold to categorize the sample into a “low” and “high” group, which resulted in ≤7 as “low” and ≥8 as “high” life satisfaction. Health perception was assessed with the question “Would you say your health is …?” with response options “poor”, “fair”, “good” and “excellent”. This measure has been found to be associated with health behaviours and risk taking among adolescents, [35, 36]. Due to the design of the response alternatives, as well as suggested in other research [37], the answers were recoded into fair/poor vs. good/excellent.

Two different measures of SES, included in the HBSC questionnaire, were chosen as independent variables for this study. First, a single item question “how would you describe the economic situation in your family?” was used to represent “subjective SES” with response alternatives: “not at all well off”, “not so well off”, “average”, “quite well off” and “very well off”. Responses were given numerical values from 1 to 5, where 1 equalled “not at all well off” and 5 equalled “very well off”. The measure is easily understood and has been used in other research to explore associations between SES and self-rated health among adolescents [3, 6, 27, 38]. Second, the Family Affluence Scale (FAS) was used as a measure of objective SES. It was developed for use in the HBSC survey and has been validated as a measure of family wealth among school children [23]. It comprises four questions about material assets and habits, “Does your family own a car?”, “Do you have your own bedroom?”, “During the last 12 months, how many times did you travel or go on vacation with your family?”, “How many computers does your family own”. Assignment of points depending on the answer ranges from 0 to 1 to 0-3, giving a summarized range of 0-9. FAS has previously shown to be reliable and in agreement with information supplied by parents on SES [23]. Since adolescents themselves are the only source of information in the HBSC-survey, FAS was in this study treated as a proxy for objective SES.

### Statistical procedures and analysis

Statistical analyses in this study were performed using SPSS, version 20.0. Descriptive statistics are presented in terms of count and percentages for categorical variables (confounders) and means and standard deviations (SD) for continuous variables (SES-variables). Significance was assumed at *p* < 0.05 and all tests were two-sided. Dichotomization of the dependent self-rated health variables was maintained throughout analyses. Gender, age and survey year were treated as confounders. To assess correlation between the two SES-variables, Pearson’s correlation coefficient was calculated. Pearson correlation was also calculated between the three self-rated health variables to test whether they reflect different dimensions of health. The trend for inequalities in self-rated health by SES between 2002 and 2014 was investigated descriptively by calculating mean values and SD of Subjective SES and FAS between groups, below and above cut-off points, for the three health measures. Independent samples t-tests were then conducted for each survey-year to reveal the significance of observed differences between groups.

In the logistics regression procedure, crude analyses were first conducted for independent variables and confounders. Odds ratio (OR) with confidence interval (95%CI) was used as an index of effect size to demonstrate increased risk for negative health outcomes. After the initial testing, FAS and confounders were entered in the first model, followed by adding subjective SES in the second model. In the third and fourth model, we added two-way interaction by cross-product terms between gender and the two SES-variables, one at a time. We considered Nagelkerke R Square to ensure the best model fit to the data. To deal with potential problems arising from comparison across the four models, we applied the method of Benjamini & Hochberg on all *p*-values in the final models to control the false discovery rate at 5% [39]. Adjusted significance levels were set to; MHC-model: *p* < 0.0416, life satisfaction-model: p < 0.0416 and health perception-model: *p* < 0.0409.

## Results

Descriptive statistics are displayed in Table 1. Girls were overrepresented in MHC, low life satisfaction and poor/fair health perception compared to boys. Also, the percentage of adolescents who reported MHC, low life satisfaction and poor/fair health perception was highest among 15-year olds. The percentage of adolescents that reported MHC and low life satisfaction was higher in 2014 compared to the other survey years. In contrast, the percentage of adolescents that rated poor/fair health perception was lowest in 2014. Regarding the SES-variables, the total mean value for subjective SES was 4.17 (SD = 0.86), on a range from 1 to 5. Furthermore, the mean values for both FAS and subjective SES were slightly lower in the groups that reported MHC, low life satisfaction and poor/fair health perception, compared with the total sample.

**Table 1:** Descriptive statistics (frequency, means and SD) of included variables in the study (*n* = 23,088)

	Total		Multiple health complaints		Low life satisfaction (≤7)		Low health perception (Poor/Fair)	
	(n = 23,088)		(*n* = 7357)		(*n* = 9180)		(*n* = 2596)	
	n	(%)	n	(%)	n	(%)	n	(%)
*Gender*								
Boys	11,461	(49.9)	2734	(23.9)	4018	(35.1)	997	(8.7)
Girls	11,486	(50.1)	4591	(40.0)	5104	(44.4)	1583	(13.7)
Total	22,947	(100,0)	7325	(31.9)	9122	(39.8)	2580	(11.2)
*Age*								
11-year olds	8054	(34.9)	1938	(24.1)	2153	(26.7)	580	(7.2)
13-year olds	7266	(31.5)	2348	(32.3)	2976	(41.0)	885	(12.2)
15-year olds	7768	(33.6)	3071	(39.5)	4051	(52.1)	1131	(14.6)
Total	23,088	(100.0)	7357	(31.9)	9180	(39.8)	2596	(11.2)
*Year*								
2002	3926	(17.0)	1327	(33.8)	1542	(39.3)	505	(12.8)
2006	4415	(19.1)	1297	(29.4)	1522	(34.5)	522	(11.8)
2010	6880	(29.8)	1990	(28.9)	2545	(37.0)	895	(13.0)
2014	7867	(34.1)	2743	(34.9)	3571	(45.4)	674	(8.6)
Total	23,088	(100.0)	7357	(31.9)	9180	(39.8)	2596	(11.2)
*FAS (0-9)*								
Mean (SD)	6.28	(1.67)	6.20	(1.70)	6.14	(1.68)	5.94	(1.75)
*Subjective SES (1-5)*								
Mean (SD)	4.17	(0.86)	3.93	(0.97)	3.85	(0.93)	3.75	(1.04)

*FAS* Family affluence scale

Pearson’s correlation coefficient was calculated between FAS and subjective SES, showing a value of 0.259. Comparing across the three health outcomes, the correlation between MHC and life satisfaction was the strongest. The health perception measure had a weaker correlation compared to the other two outcomes (see Table 2).

**Table 2:** Pearson´s correlation coefficients (r) between the three health outcomes

	Life satisfaction	Health perception	Multiple health complaints
	Pearson’s r	Pearson’s r	Pearson’s r
Multiple health complaints	0.319	*	*
Life satisfaction	*	0.230	*
Health perception	*	*	0.231

* indicates duplicate analyses

**Fig. 1:**
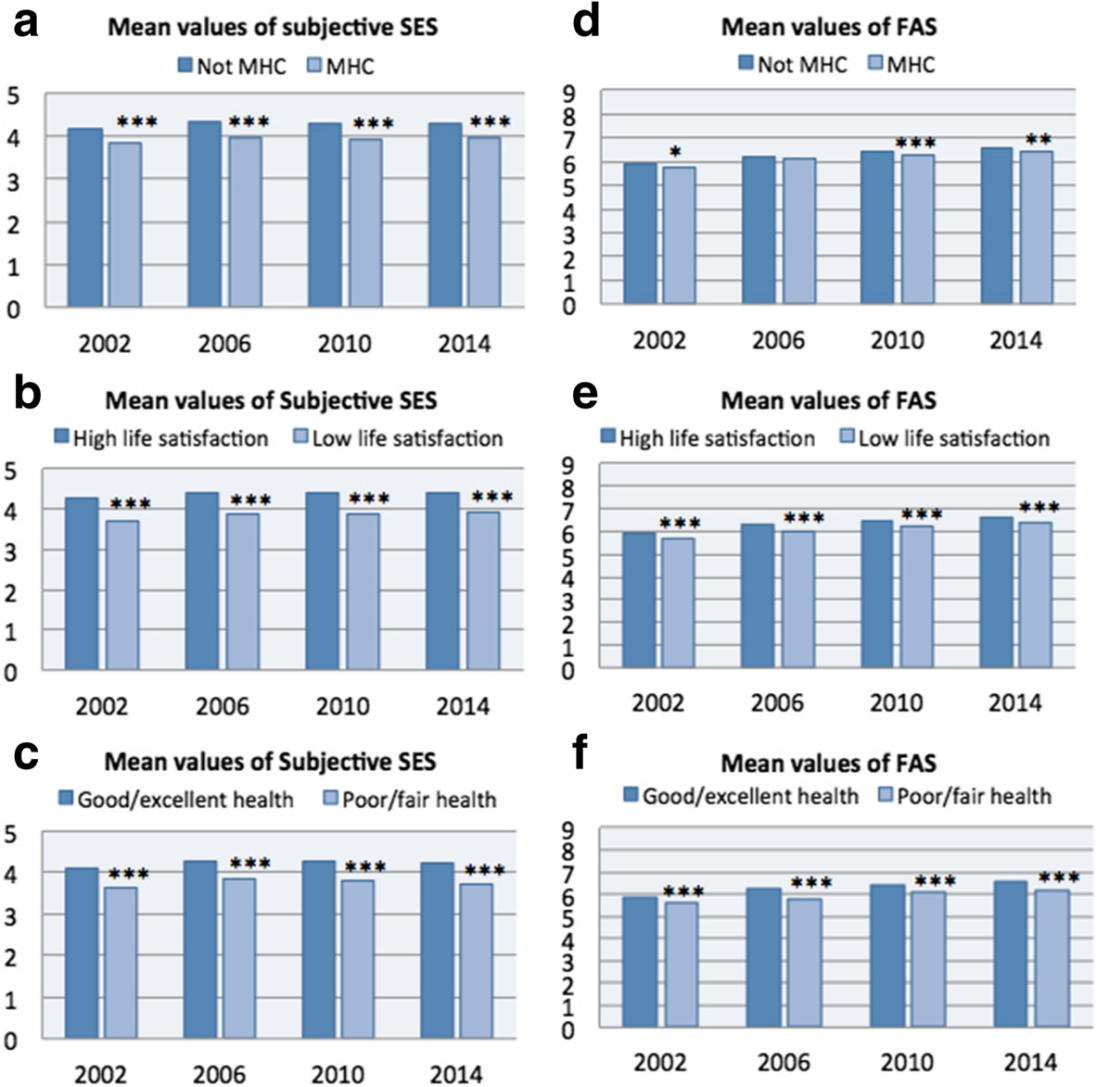
Descriptive trends showing mean values of subjective SES and FAS for groups above and below cut-off points of health outcomes by each survey year. Independent t-tests to show significant inequalities between groups. * *p* = <0.05 ** *p* = <0.01 *** *p* = <0.001

Figure 1 (a-f) shows descriptively the inequalities observed for each health outcome by both the subjective and objective measures of SES for each survey year (using the cut-off points highlighted in the methods section). For subjective SES, mean values remained fairly constant over time in both groups of each health measure (Fig. 1a-c). Additionally, the gap between the two groups was significant in every survey year between 2002 and 2014 for MHC, life satisfaction and health perception (*p* < 0.001). Unlike subjective SES, a linear increase was found in mean values of FAS over time. Inequalities in FAS between adolescents that reported not MHC (below the cut-off point) and those who reported MHC varied over time, the lowest in 2006 (*p* = 0.115) and the highest in 2010 (p < 0.001) (Fig. 1d). For life satisfaction and health perception, inequalities by FAS varied somewhat over time but remained significant between 2002 and 2014 (p < 0.001) (Fig. 1e and f).

A first step in the logistic regression modelling was to investigate missing cases for the ultimate models to identify possible non-random patterns. Missing cases were found to be distributed with a 6:4 ratio between boys and girls. The majority of adolescents that failed to report their SES through FAS or subjective SES, reported not MHC (61%), high life satisfaction (56%) and good/excellent health perception (86%). The group of adolescents that failed to report health complaints, life satisfaction or health perception, were observed to have a slightly higher mean value of subjective SES compared to the total sample, in contrast to FAS where a slightly lower mean value was observed. The missing cases in the three logistic regression models were: *n* = 1813 or 7.9% (health perception);, *n* = 2033 or 8.8% (life satisfaction); and *n* = 2252 or 9.8% (MHC).

Multiple logistic regression analyses are presented in Table 3. In the first model, higher FAS-scores were found negatively related to MHC, low life satisfaction and low perceived health. When subjective SES was added in the second model, effect sizes were weakened for FAS in relation to life satisfaction (OR: 0.98, CI: 0.97; 1.00) and health perception (OR: 0.96, CI: 0.93;0.98) and even indicated a weak positive relationship to MHC (OR: 1.02, CI: 1.00;1.04). On the other hand, effect sizes indicated that higher subjective SES was negatively related to MHC, low life satisfaction and low perceived health (Table 3). In models 3 and 4, interaction terms for gender with FAS and gender with subjective SES were entered, one at a time. Interactions for gender with FAS indicated no gender differences in the relation between FAS and MHC, life satisfaction and health perception. However in model 4, gender with subjective SES indicated significant gender differences in the relation between subjective SES and MHC (*p* = 0.030) and life satisfaction (*p* = 0.021). This suggested a stronger negative relationship between higher subjective SES and MHC and life satisfaction for girls than for boys.

**Table 3:** Multiple logistics regression models, odds ratios with 95% confidence interval (95%CI) of health by family affluence and subjective SES

	Multiple health complaints			Low life satisfaction (≤7)			Low health perception (Poor/Fair)		
	OR	(95%CI)	*p* ^a^	OR	(95%CI)	*p* ^a^	OR	(95%CI)	*p* ^a^
***Crude***									
FAS (0-9)	**0.96**	(0.94;0.98)		**0.92**	(0.90;0.93)		**0.87**	(0.85;0.89)	
Subjective SES (1-5)	**0.63**	(0.61;0.65)		**0.47**	(0.45;0.48)		**0.57**	(0.55;0.60)	
Gender (girls)	**2.09**	(1.97;2.21)		**1.48**	(1.40;1.56)		**1.67**	(1.54;1.82)	
Age									
*—11 year olds*	1.00	(reference)		1.00	(reference)		1.00	(reference)	
*—13 year olds*	**1.46**	(1.36;1.57)		**1.94**	(1.81;2.08)		**1.79**	(1.60;1.99)	
*—15 year olds*	**1.98**	(1.85;2.12)		**3.05**	(2.85;3.26)		**2.19**	(1.97;2.43)	
Survey year									
*2002*	1.00	(reference)		1.00	(reference)		1.00	(reference)	
*2006*	**0.80**	(0.73;0.88)		**0.82**	(0.75;0.89)		0.91	(0.80;1.04)	
*2010*	**0.81**	(0.74;0.88)		0.95	(0.88;1.03)		1.03	(0.92;1.16)	
*2014*	1.05	(0.97;1.14)		**1.34**	(1.24;1.45)		**0.64**	(0.57;0.73)	
***Model 1*** ^**b**^									
FAS (0-9)	**0.96**	(0.94;0.98)		**0.90**	(0.89;0.92)		**0.89**	(0.87;0.91)	
Nagelkerke		0.074			0.100			0.048	
***Model 2*** ^**b**^									
FAS (0-9)	**1.02**	(1.00;1.04)		0.98	(0.97;1.00)		**0.96**	(0.93;0.98)	
Subjective SES (1-5)	**0.66**	(0.63;0.68)		**0.51**	(0.49;0.53)		**0.62**	(0.59;0.65)	
Nagelkerke		0.107			0.176			0.081	
***Model 3*** ^**b**^									
FAS (0-9)	**1.03**	(1.00;1.06)		0.99	(0.96;1.02)		**0.95**	(0.92;0.99)	
Subjective SES (1-5)	**0.66**	(0.63;0.68)		**0.51**	(0.49;0.53)		**0.62**	(0.59;0.65)	
Gender*FAS			0.387			0.535			0.897
Nagelkerke		0.107			0.176			0.081	
***Model 4*** ^**b**^									
FAS (0-9)	**1.02**	(1.00;1.04)		0.98	(0.97;1.00)		**0.96**	(0.93;0.98)	
Subjective SES (1-5)	**0.68**	(0.65;0.72)		**0.53**	(0.50;0.56)		**0.64**	(0.59;0.69)	
Gender*subjective SES			0.030			0.021			0.380
Nagelkerke		0.107			0.177			0.081	

^a^ Significance level of interaction for gender by SES

^b^ Model was adjusted for age, gender and survey-year

Bold **OR**:s indicate significance at the corrected level by the Benjamini & Hochberg procedure, MHC: *p* < 0.0416, Life satisfaction: *p* < 0.0416, General health perception: *p* < 0.0409

*FAS* Family affluence scale

## Discussion

This study investigated socioeconomic inequalities in adolescent self-rated health among Swedish adolescents. One of our objectives was to investigate how inequalities were portrayed through different measures of SES, thereby showing the complexity of methodology. We found that a subjective measure of SES, in this study portrayed by the perception of the familial economic situation, revealed contrasting results to those of the objective measure, portrayed by FAS. Initially, our results suggested a modest but statistically significant social gradient in adolescent health when assessed by the objective measure. When the two SES-variables were entered simultaneously, the relationship between FAS and health outcomes was weakened, while a stronger relationship between subjective SES and health was evident. Similar results have been found in research targeting other countries concluding that health inequalities by subjective measures still existed after controlling for material assets [40].

Even though not all is known about the circumstances surrounding the relationship between subjective SES and adolescent health, some authors have speculated on plausible explanations to its predictive capabilities. For example, it may be that although the perception of family SES is influenced by objective SES-markers, relative comparisons within groups may have a stronger impact on adolescents´ self-image than actual societal standing [41]. It could also be that subjective SES is also likely to involve the individuals’ feelings regarding past events and attitude towards the future, potentially accounting for experience and developmental disparities between adolescents [26, 41]. The bidirectional influence between subjective SES and health outcomes should also be considered. There is a possibility that MHC, low life satisfaction or poor/fair health perception can influence an adolescent’s perception of their familial economic situation negatively. Notwithstanding these considerations our results demonstrate that subjective measures of SES contribute an important dimension to our understanding of inequalities in adolescent health. The inclusion of such measures is particularly important in health surveys of adolescents since their ability to report the socio-economic status of their parents (by stating their occupation) has shown to be poor [15].

This study chose to include a range of different outcome measures to reflect both positive and negative aspects of adolescent health. In this regard, it is interesting to note that MHC, life satisfaction and health perception behaved similarly in in their respective relationship with the two SES-variables. When FAS was entered separately in analyses, the relation between MHC and FAS was slightly weaker than of life satisfaction, health perception and FAS. Other studies have found similar patterns of relationship. Specifically that the socioeconomic gradient for MHC tends to be less apparent than for life satisfaction and health perception [19, 37]. Our findings showed however that this was also true for the subjective measure of SES. It’s inclusion in the analysis demonstrated that the relationship between MHC and FAS was stronger than that observed for the other two health outcome measures (life satisfaction and health perception). When subjective SES was entered in the final analysis, higher FAS-scores appeared to be related to the occurrence of MHC. This is contradictory to the well-documented relationship between SES and health documented elsewhere [42].

We investigated if there were any visible trends in socioeconomic inequalities in health between 2002 and 2014. When assessed by subjective SES, inequalities in MHC, life satisfaction and health perception remained significant with little variation between 2002 and 2014. When assessed by FAS, inequalities in MHC increased between 2006 and 2010 and remained significant in 2014. Inequalities by FAS in adolescent well-being have previously been found to be greater in countries with more unequal income-distribution [4]. Thus, if income inequality continues to increase in Sweden, it could be argued that a consequential development is an increase in socioeconomic health inequalities amongst this age group. However, trends can be divergent depending on how SES is assessed. If SES is measured subjectively, increased socioeconomic health inequalities might not be visible. A European study that investigated socioeconomic health inequalities among adolescent by subjective SES between 1994 and 2010 found that inequalities remained constant in most countries included in the analysis [3]. Another study looking at trends between 2002 and 2010, that included many of the same countries, found increased socioeconomic inequalities in many aspects of adolescent health, when FAS was used as an indicator of SES [43]. To summarize, while health inequalities by subjective measures remain at consistently high levels, socioeconomic inequalities assessed through objective measures are increasing, although this was only partially supported in our study of Swedish adolescents. Continued monitoring of the development of these inequalities is necessary to support policy makers to steer their efforts appropriately using evidence from research. Our inability to understand the precise links between SES and health amongst adolescents may have future social and economic consequences. Such evidence can steer more action to preventative efforts at the political level.

## Limitations

There are a number of limitations. Firstly, due to the cross-sectional method of HBSC-surveys, causal mechanisms associated with our findings could not be made. This is a common limitation of such studies, which can only be overcome by longitudinal design. Over and above this drawback, the contribution of HBSC is crucial to cross-national comparisons as well as mapping of adolescent health within countries [44]. Secondly, dichotomization of the included health-variables in this study may be considered a weakness. We are aware of the potential loss of information that this manipulation of data can bring as well as the risks with “norming”, i.e. setting the threshold based on the proportion of the sample falling above or below that threshold [45]. In an attempt to lessen this weakness, cut-off points were drawn from recommendations made by the HBSC-network and previous literature [19, 46]. That said, the threshold that we used to distinguish between “low” and “high” life satisfaction was set fairly high due to the high mean value of the sample. We recognize that this might make future cross-national comparisons more difficult. It has been argued by antagonists of dichotomization of variables that large sample sizes can minimize the potential loss of power in such analyses [47] and our study was based on a fairly large sample. A third weakness relates to the proportion of missing cases in the final regression model. Missing cases ranged from 7.9 to 9.8% as a result of missing responses in one or more of the included variables. In an attempt to understand the influence of missing cases on our findings, we examined possible non-random patterns. We found no strong patterns that suggest adolescents with low SES or poor health to be overrepresented among the missing cases. Boys represented about 60% of the missing cases compared to 40% girls. Therefore we argue it is unlikely that our findings were strongly impacted by missing cases. Lastly, the mean value for subjective SES of the total sample was surprisingly high, 4.17 (SD: 0.86) on the 1-5 scale. This might be a consequence of the design of response alternatives where “average” is assumed to be something other than “quite well off” and “very well off”, depending on the understanding of what “average” implies. Adolescents may neglect that scoring above “average” equals to be better off than the average of Swedish adolescents. By treating the variable as continuous in analyses, we reduced the risk of this potentially affecting our results.

## Conclusions

Overall, this study showed that socioeconomic inequalities in adolescent health defined by subjective SES were larger than when assessed objectively by material assets. We conclude that the level of inequalities varied depending on how SES was being measured and that subjective SES contributes to a deeper understanding of health inequalities. This study also found that inequalities as expressed by the two SES-variables were similarly portrayed across MHC, life satisfaction and health perception. Our findings show that in this Swedish population, subjective socioeconomic inequalities in health have remained stable between 2002 and 2014. However, when SES was measured objectively, we found some indications toward increased inequalities in multiple health complaints over the same time period. Our findings imply that approaching SES from a subjective perspective is a necessary supplement to the objective approach as it can provide a more rounded picture of socioeconomic inequalities in adolescent health. We do however recognise that further validation studies are needed to expand the range of robust measures available to research to capture the subjective and objective domain. Additionally, similar studies carried out in different country contexts are required to substantiate our findings.

This study should be useful to policy makers interested in promoting equity in adolescent health as it gives them an opportunity to reflect on the types of strategies that would alleviate problems associated with subjective levels of inequalities. Importantly, ensuring that a range of strategies is employed that tackle both material and wider social factors known to impact on health.

## Abbreviations

FAS: Family Affluence Scale
GDP: Gross domestic product
HBSC: Health Behaviour of School-aged Children
MHC: Multiple health complaints
SCL: Symptom checklist
SES: Socioeconomic status
SPSS: Statistical package for social sciences
